# Research note: Stress responses in different genetic strains of laying hens housed in a cage-free environment^☆^

**DOI:** 10.1016/j.psj.2025.105295

**Published:** 2025-05-15

**Authors:** Bhavisha P. Gulabrai, Allison N. Pullin, Kenneth E. Anderson, Aaron S. Kiess

**Affiliations:** Prestage Department of Poultry Science, College of Agriculture and Life Sciences, North Carolina State University, Raleigh, NC, 27607, USA

**Keywords:** laying hen, Cage-free, Stress, Corticosterone

## Abstract

Cage-free systems offer hens the opportunity to perform natural behaviors, but they also expose birds to more frequent environmental and social stressors, which can impact welfare. Therefore, this study assessed stress responses in four laying hen strains housed in a cage-free environment. Hy-Line W36 White, H&N White, Hy-Line Brown, and Bovan Brown laying hens were evaluated through plasma corticosterone levels, heterophil lymphocyte ratios, and heat shock proteins (HSP) 70, 90a, and 90b gene expression at five distinct time points throughout the laying hen cycle. There were no differences among these parameters found attributable to genetic strain. However, all strains exhibited similar stress responses at critical time points throughout the lay cycle. HSP70 expression was highest at the end of lay (*p* < 0.0001), HSP90a at the start of lay (*p* < 0.0001), and both HSP90b and heterophil lymphocyte ratios at peak lay (*p* < 0.0001), suggesting a potential relationship between the two. Corticosterone levels remained consistent across both genetic strains and time points. Therefore, although no distinctions were observed among genetic strains, the variability in stress levels over time provides insight into factors impacting the welfare of hens throughout egg production.

## Introduction

In the United States laying hen industry, several factors have contributed to a shift towards cage-free production. One of the main elements of concern is ensuring that hens can exhibit a full behavioral repertoire (Van Goor et al., 2020). While cage-free systems support species-specific behaviors, challenges remain regarding efficiency and management in these environments. Due to the complexity and dynamics of cage-free environments, it is not uncommon for laying hens to experience more frequent sudden stressors (Nelson et al., 2020). Environmental and social stressors are characteristics of cage-free environments that may cause distress or negatively impact the performance, behavior, and overall welfare of laying hens (Van Goor et al., 2020). Genetic strains that can cope with these stressors more successfully may be better suited for cage-free production (Brown et al., 2022).

Stress responses activate either the sympathetic-adrenal-medullary (SAM) axis, or the hypothalamic-pituitary-adrenal (HPA) axis. Glucocorticoids are stress hormones released by the HPA axis, and the primary avian glucocorticoid, referred to as the stress hormone, is corticosterone (CORT) (Singh et al., 2009). CORT is an indicator of the short-term coping strategies of laying hens, plays a key role in energy homeostasis, particularly during the metabolically demanding process of egg production, and can yield long-term effects on overall performance (Singh et al., 2009). CORT is a widespread tool utilized to measure stress responses, specifically in blood serum or plasma (Kang and Shim, 2021). Based on previous research, it is evident that CORT concentrations differ amongst various genetic strains. Nelson et al. (2020) demonstrated differences between and within white and brown breeds, Brown et al. (2022) identified higher concentrations in brown strains compared to white strains, while Pusch et al. (2018) found no differences between brown and white layers in response to acute stressors. Therefore, various genetic strains may utilize different physiological mechanisms to cope with stressors. Other common metrics of physiological stress are heterophil lymphocyte (H:L) ratios, which are strongly linked to CORT levels (Van Goor et al., 2020), and the upregulation of heat shock proteins (HSP), which also impact growth and performance (Kang and Shim, 2021).

H:L ratios are blood markers of long-term stress (Singh et al., 2009). In the event of a stressor, the number of heterophil cells increase in the bloodstream, while the number of lymphocytes decrease, resulting in an overall increase of the heterophil to lymphocyte ratio (Pusch et al., 2018). This ratio is calculated by counting a combined total of 100 heterophils and lymphocytes (Singh et al., 2009). Singh et al. (2009) validated the utilization of H:L ratios as indicators of chronic stress as overall ratios increased over time. Previous research demonstrates brown strains of laying hens exhibiting higher ratios when compared to white strains (Singh et al., 2009; Pusch et al., 2018; Brown et al., 2022). While there is a positively correlated relationship between elevated glucocorticoid production and circulating heterophils, both indicators represent different durations of stress.

HSPs are polypeptides induced by heat that may either be continuously expressed or caused by stress (Murugesan et al., 2017). Stressful events cause proteins to become impaired and unfold (Murugesan et al., 2017). This prompts the accelerated production of HSPs as they link to proteins that have unfolded, aiding in protein structure and function (Kang and Shim, 2021). High egg production has the potential to cause an increase in metabolic heat in laying hens and therefore HSP expression (Murugesan et al., 2017). Aside from heat stress, an elevation in HSP production can be due to a variety of stressors such as human contact, disease, and injury (Pusch et al., 2018). HSP70 and HSP90 tend to be the most established indicators used in literature (Xie et al., 2014).

The primary function of HSP70 involves reactivating proteins that have unfolded, while HSP90 stabilizes proteins in a stress response (Xie et al., 2014). Xie et al. (2014) and Kang & Shim (2021) additionally noted increases of HSP70 during acute heat stress and HSP90 during chronic heat stress. In response to heat stress, previous research has determined differences in HSP70 and HSP90 expression within and between white and brown genetic strains of laying hens (Pusch et al., 2018). HSP90 is further categorized into HSP90 alpha (HSP90a) and HSP90 beta (HSP90b). The functional differences between the two have only been determined in mammals, but based on those findings, HSP90a is induced in the event of a stressor while HSP90b is constitutively expressed (Hoter et al., 2018).

Various physiological mechanisms may be utilized by different genetic strains of laying hens in their response to stress. Typically, white strains of laying hens have been selectively bred to excel in conventional cage environments (Brown et al., 2022), potentially rendering them less suitable for cage-free production (Nelson et al., 2020). Therefore, the objective of this study was to determine if genetic strain influenced stress parameters in a cage-free environment. It was hypothesized that stress levels would be higher in white laying hens compared to brown laying hens due to their inability to cope with environmental and social stressors.

## Materials and methods

This study was conducted at North Carolina State University’s Poultry Teaching Unit in Raleigh, NC and approved by the North Carolina State University Institutional Animal Care and Use Committee (IACUC #22-219). Industry sources provided two white commercial strains of laying hens, Hy-Line W36 White and H&N White, and two brown commercial strains of laying hens, Hy-Line Brown and Bovan Brown for this trial. Prior to placement at our facility, pullets were reared as follows: Hy-Line W-36 White pullets were reared in cages to 11 weeks of age (WOA), H&N White pullets were reared on cage-free wire flooring to 11 WOA, Hy-Line Brown pullets were reared on cage-free litter flooring to 11 WOA, and Bovan Brown pullets were reared on cage-free litter flooring to 9 WOA. At placement in our facility, pullets were wing-tagged with identification numbers and distributed amongst twelve 3.2 × 1.2 m cage-free floor pens containing litter, one feeder, one bell drinker, one 3-rung ladder perch, one roost, and four nest boxes per pen. Perches were provided at 8.88 inches of linear space per bird. For each pullet strain, 75 birds were distributed amongst 3 replicate pens (4 strains X 75 birds/strain = 300 birds total). A total of 3 birds/pen were randomly selected for sampling and identified by blue leg bands.

Physiological stress data was evaluated at five different time points throughout the lay cycle: pullet (17 weeks of age (WOA)), start of lay (22 WOA), peak lay (32 WOA), mid lay (50 WOA), and end of lay (70 WOA). Parameters included HSPs 70, 90a, and 90b gene expression in the liver, plasma CORT concentrations, and H:L ratios. All parameters were collected at a consistent time of day between 7:00 AM and 10:00 AM across all strains and ages. HSP gene expression was analyzed in the liver as this organ is more susceptible to heat stress than other organs (Murugesan et al., 2017). However, HSPs upregulate in the event of other stressors that cause proteins to unfold such as handling, disease, and injury. HSP70 assists in reactivating unfolded proteins and is linked to acute heat stress, while HSP90 aids in protein stabilization and linked to chronic heat stress. In mammals, HSP90a is upregulated in the event of a stressor and HSP90b is regularly expressed (Hoter et al., 2018). CORT is the major stress hormone in chickens, and a common indicator of short-term stress (Singh et al., 2009). H:L ratios are an additional hematological measure in which the number of heterophils and lymphocytes are counted to a total of 100 cells. Higher ratios are indicators of long-term stress.

### Heat shock protein gene expression

One hen/pen, or 3 hens/treatment, were utilized to obtain HSP gene expression. Hens were euthanized via cervical dislocation, and a small portion of the left lobe of the liver, no thicker than 0.5 cm, was preserved in 1.3 ml of RNA*later*. RNA was extracted using a maximum of 30 mg of liver tissue using an RNeasy Mini Kit (QIAGEN, Hilden Germany) following manufacturer’s instructions. Briefly, liver samples were homogenized in supplied buffer RLT to lyse cells and then centrifuged to separate debris. The supernatant was transferred to a new tube containing 70 % ethanol, mixed, then transferred to a spin column to be centrifuged. A series of washes using RW1 and RPE buffers were performed to remove excess ethanol. RNA was then eluted from the column using nuclease-free water. RNA was quantified using the Nanodrop ND2000 spectrophotometer (Thermo Fisher Scientific, Waltham, Massachusetts, USA). RNA was reverse transcribed to cDNA using a High-Capacity cDNA Reverse Transcription Kit (Applied Biosystems™ Waltham, Massachusetts, USA) for use in quantitative polymerase chain reaction (qPCR).

Gene expression was assessed using qPCR run in triplicate reactions. Each 20 μl reaction contained 2.5 ng cDNA template, 2X Power SYBR Green PCR Master Mix (Applied Biosystems™ Waltham, Massachusetts, USA), and 500 nM forward and reverse gene-specific primer. Primers used are represented in Table 1. PCR was run on the StepOnePlus real time PCR system (Applied Biosystems™ Waltham, Massachusetts, USA). The initial denaturation process was run on one cycle at 95 °C for 10 minutes. Denaturation, annealing, and extension were repeated for 40 cycles at 95 °C for 30 seconds, 60 °C for 30 s, and 72 °C for 30 seconds, respectively. HSP gene expression were normalized to Beta Actin expression.

**Table 1:** Primers used for heat shock protein gene expression^1^.

Sequence name	Sequence
HSP70-F	GCG GAG CGA GTG GCT GAC TG
HSP70-R	CGG TTC CCC TGG TCG TTG GC
HSP90AA-F	CGA CCA ACC AAT GGA GGA GG
HSP90AA-R	TGA TCT TGT CCA GAG CAT CAG A
HSP90B-F	GGC TCT GGC ATG CAC GCT TC
HSP90B-R	TCT TCC ACG GTC GCA TCC ACA
Bact-F	GTC CAC CTT CCA GCA GAT GT
Bact-R	ATA AAG CCA TGC CAA TCT CG

1 Table 1 demonstrates the forward and reverse primers used for heat shock proteins 70, 90a, and 90b as well as the housekeeping gene, Beta Actin.

### Corticosterone

A total of 3 hens/pen, or 9 hens/treatment, were utilized to determine plasma CORT concentrations. Approximately 3 ml of blood were obtained via the brachial vein approximately 45 seconds after catching and poured into an EDTA blood tube. Blood tubes were immediately placed on a Labquake Shaker blood tube rotator (Labindustries INC., Berkeley, California) to prevent clotting, and then into a cooler with ice for transport. Tubes were then centrifuged at 8 °C at 3000 rpm for 20 min, and plasma was transferred into microcentrifuge tubes. Samples were frozen at −20 °C until analyzed using a corticosterone competitive enzyme-linked immunosorbent assay (ELISA) kit (Thermo Fisher Scientific, Waltham, Massachusetts, USA) according to manual procedures. In short, 5 μl of dissociation reagent was added to microcentrifuge tubes followed by the addition of 5 μl of plasma samples. The sample was diluted with assay buffer, and standards underwent a series of dilutions. 50 μl of samples and standards were added to designated wells, followed by the addition of 25 μl each of corticosterone conjugate and corticosterone antibody. The plate was incubated at room temperature with shaking using a MaxQ 4450 Orbital Shaker (Thermo Fisher Scientific, Marietta, Ohio) for one hour. After incubation, the solution was aspirated, and the wells were washed. Then, 100 μl of TMB substrate was added to each well and incubated for 30 min at room temperature. Finally, 50 μl of stop solution was added to each well. Absorbance was measured using a BioTek Synergy HTX multi-mode plate reader (Agilent, Santa Clara, California). Concentrations were calculated in duplicate relative to standards.

### Heterophil lymphocyte ratios

Before blood tubes were centrifuged, 3 μl of blood were pipetted onto a microscope slide. Utilizing another sterile slide, blood was smeared across the slide and air dried for 15 min. Once dry, slides were stained with a Wrights-Giemsa stain (RICCA Chemical Company, Arlington, Texas). Slides were placed in a glass slide container and dipped into the stain for 15 s. Then, the slide container was removed from the stain and placed in deionized water for 30 s. Slides were then removed from the container and placed on a tray. Slides air dried for approximately 30 min. Once slides were stained and dried, two droplets of Permount mounting medium were placed directly onto the stained slide adhering a Corning 24 × 50 mm coverslip to each slide. After drying for 24 h, slides were viewed under a microscope at 100x magnification. A total of 100 cells were counted to achieve a H:L ratio.

### Statistical analysis

Data were analyzed with JMP Pro 17 using a two-way ANOVA and considered significant when *P* ≤ 0.05. Genetic strain and period were considered main effects. Tukey’s HSD was used to determine significant differences amongst means.

## Results and discussion

The results of physiological parameters examined in this study are represented in Table 2. Although there were no differences found amongst strains for HSP gene expression, there were varying levels of HSP expression over time. All strains had higher levels of HSP70 expression at the end of lay (*p* < 0.0001) when compared to all other time points. HSP90a expression was highest at the start of lay (*p* < 0.0001) when compared to peak, mid, and end of lay for all strains. HSP90b gene expression was higher at peak lay (*p* < 0.0001) compared to all other time points. No differences were found amongst strains or time points for CORT. All strains exhibited higher H:L ratios at peak lay (*p* < 0.0001) in comparison to all other time points.

**Table 2:** The effect of genetic strain and time point on stress physiology parameters^1^.

	HSP70	HSP90a	HSP90b	Corticosterone	H:L Ratio
Strain					
W36 White	0.857	1.115	1.053	637.970	0.628
H&N White	0.870	1.078	1.044	5374.091	0.658
Hyline Brown	0.878	1.050	1.116	4271.200	0.839
Bovan Brown	0.580	1.093	1.110	5907.215	0.615
P-value	0.4724	0.0606	0.2282	0.1465	0.5279
SEM	0.014	0.017	0.031	1744.008	0.128
Time Point					
Pullet	0.840^bc^	1.163^ab^	1.016^b^	155.703	0.559^b^
Start Lay	0.825^c^	1.200^a^	1.071^b^	3674.971	0.639^b^
Peak Lay	0.793^c^	1.098^b^	1.249^a^	4258.974	1.593^a^
Mid Lay	0.888^b^	0.954^c^	1.025^b^	5738.577	0.303^b^
End Lay	0.974^a^	1.004^c^	1.045^b^	6409.869	0.334^b^
P-value	<0.0001	<0.0001	<0.0001	0.1899	<0.0001
SEM	0.015	0.019	0.034	1961.862	0.141
Interaction					
P-value	0.2266	0.9897	0.4522	0.3863	0.7756
SEM	0.030	0.038	0.068	3899.719	0.279
CV (%)	86.404	108.393	108.113	401862.600	70.084

^a,b,c^ signifies statistical significance along columns (*P* < 0.05).

1 Table 2 provides genetic strain and time point results on heat shock protein (HSP) 70, HSP90a, and HSP90b relative normalized gene expression values in the liver, blood plasma corticosterone (CORT) concentrations, and heterophil to lymphocyte (H:L) ratios. Heat shock protein values were normalized to Beta Actin and acquired from 3 hens per treatment for each time point. The absorbance of CORT was measured in duplicate relative to standards and acquired from 9 hens per treatment. H:L ratios were calculated from counting a combined total of 100 heterophil and lymphocyte cells and acquired from 9 hens per treatment.

The primary function of HSP70 is to reactivate unfolded proteins (Xie et al., 2014). In this study, all laying hen strains expressed HSP70 in highest amounts at the end of lay. This may be due to protein unfolding caused by reproductive stress at the end of the lay cycle, but future research is needed to confirm this. The differences between HSP90a and HSP90b have yet to be explored in chickens. Nonetheless, their functions appear different as demonstrated by varying levels of expression observed at different time points in this study.

All strains expressed the highest levels of HSP90a at the start of lay compared to peak, mid, and end of lay. In mammals, HSP90a is generated in the event of a stressor (Hoter et al., 2018). At the start of lay, hens experience various stressors that may increase HSP90a expression for protein folding and growth support, with changes in reproductive demands and environmental factors potentially influencing HSP90a levels as hens age. However, further research is needed to confirm these potential relationships. Additionally, hens may experience changes in hormone levels and immune function causing increased expression especially in the liver (Murugesan et al., 2017). Although HSP90b is continuously expressed in mammals (Hoter et al., 2018), this study shows that hens expressed HSP90b in the highest amounts at peak lay. Peak lay is characterized by the highest levels of egg production which may require increased HSP90b expression (Murugesan et al., 2017). Protein folding and the functioning of HSPs appear to be essential for hens experiencing metabolic stress and peak egg production.

In this study, CORT concentrations did not differ amongst strains or time points. CORT serves as a reliable indicator of short-term stress and plays a significant role in highly metabolic processes like egg production. There is a brief time interval in which concentrations spike following the exposure to a stressor; therefore, the collection method used in this study may not have accurately captured this event. These results are consistent with Pusch et al. (2018) as no differences were found among white and brown strains. However, additional research has found differences between and within brown and white strains (Nelson et al., 2020), as well as brown strains exhibiting higher CORT levels than white strains (Brown et al., 2022). Based on previous literature, the varying results found in CORT concentrations between brown and white laying hen strains provide insight into the contributions of genetic and methodological factors. Our study’s inability to determine differences between white and brown strains suggests a need for further research on laying hen stress responses and genetic predispositions.

All strains experienced the highest H:L ratios at peak lay compared to all other time points. Hens typically reach peak egg production at 32 weeks of age causing intense reproductive activity. Long-term stress may be induced as a hen’s reproductive system experiences ongoing elevated physiological demand. Al-Murrani et al. (2006) demonstrated a relationship between H:L ratios and egg production/reproduction. Furthermore, previous research has found strain differences where brown strains of laying hens demonstrated higher H:L ratios in comparison to white strains (Singh et al., 2009; Pusch et al., 2018; Brown et al., 2022). In conclusion, our study demonstrated all strains having higher H:L ratios at peak lay, aligning with previous research indicating a correlation between H:L ratios and high egg production. However, unlike other studies where brown strains exhibited higher ratios compared to white strains, this study did not observe any strain differences. Further research exploring factors that influence H:L ratios among different laying hen strains may provide additional insight into the relationship between stress responses and the lay cycle.

Our findings highlight the dynamic expression of HSP gene expression and stress-related parameters throughout the lay cycle, while also emphasizing the need for further investigation into genetic influences on stress responses. Future research should refine methodologies and explore additional physiological markers to enhance our understanding of stress adaptation in cage-free production systems.

## Funding

This research received funding from the Animal Health and Nutrition Consortium at 10.13039/100007703North Carolina State University [grant number 000046].

## Declaration of competing interest

The corresponding author, on behalf of all the authors of this submission, declare that there are no conflict of interests that must be disclosed which could inappropriately influence this work.
